# Identifying Where REDD+ Financially Out-Competes Oil Palm in Floodplain Landscapes Using a Fine-Scale Approach

**DOI:** 10.1371/journal.pone.0156481

**Published:** 2016-06-08

**Authors:** Nicola K. Abram, Douglas C. MacMillan, Panteleimon Xofis, Marc Ancrenaz, Joseph Tzanopoulos, Robert Ong, Benoit Goossens, Lian Pin Koh, Christian Del Valle, Lucy Peter, Alexandra C. Morel, Isabelle Lackman, Robin Chung, Harjinder Kler, Laurentius Ambu, William Baya, Andrew T. Knight

**Affiliations:** 1 Living Landscape Alliance, 110 Maui Court, Waikiki Condominium, Jalan Aru, Tanjung Aru, 88100, Kota Kinabalu, Sabah, Malaysia; 2 ARC Centre of Excellence for Environmental Decisions, University of Queensland, Brisbane, QLD 4072, Australia; 3 HUTAN/Kinabatangan Orang-utan Conservation Programme, 88874, Kota Kinabalu, Sabah, Malaysia; 4 Durrell Institute for Conservation and Ecology, School of Anthropology and Conservation, Marlowe Building, University of Kent, Canterbury, Kent, United Kingdom; 5 Borneo Futures initiative, Ciputat, 15412, Jakarta, Indonesia; 6 Department of Forestry and Management of Natural Environment, Technological Education Institute of Kavala, GR 61100, Drama, Greece; 7 North England Zoological Society, Research Fellow, Chester, United Kingdom; 8 Sabah Wildlife Department, Wisma Muis, 88100, Kota Kinabalu, Sabah, Malaysia; 9 Forest Research Centre, Sabah Forestry Department, P.O. Box 1407, 90715, Sandakan, Sabah, Malaysia; 10 Danau Girang Field Centre, c/o Sabah Wildlife Department, Wisma Muis, 88100, Kota Kinabalu, Sabah, Malaysia; 11 Organisms and Environment Division, School of Biosciences, Cardiff University, Sir Martin Evans Building, Museum Avenue, Cardiff, CF10 3AX, United Kingdom; 12 School of Earth and Environmental Sciences, University of Adelaide, Adelaide, South Australia, 5005, Australia; 13 Althelia ecosphere, Ecosphere Capital Limited, 1 Lumley Street, London, W1K 6TT, United Kingdom; 14 School of Geography and the Environment, University of Oxford, Oxford, OX1 3QY, United Kingdom; 15 C H Williams, Talhar and Wong (Sabah) Sdn Bhd, 90715, Sandakan, Sabah, Malaysia; 16 Borneo Conservation Trust, 5th Floor, Block B, Wisma Muis, 88100, Kota Kinabalu, Sabah, Malaysia; 17 Department of Life Sciences, Imperial College London, Silwood Park Campus, Buckhurst Road, Ascot, Berkshire, SL5 7PY, United Kingdom; 18 Department of Botany, Nelson Mandela Metropolitan University, P.O. Box 77000, Port Elizabeth, 6031, Eastern Cape, South Africa; Ohio University, UNITED STATES

## Abstract

Reducing Emissions from Deforestation and forest Degradation (REDD+) aims to avoid forest conversion to alternative land-uses through financial incentives. Oil-palm has high opportunity costs, which according to current literature questions the financial competitiveness of REDD+ in tropical lowlands. To understand this more, we undertook regional fine-scale and coarse-scale analyses (through carbon mapping and economic modelling) to assess the financial viability of REDD+ in safeguarding unprotected forest (30,173 ha) in the Lower Kinabatangan floodplain in Malaysian Borneo. Results estimate 4.7 million metric tons of carbon (MgC) in unprotected forest, with 64% allocated for oil-palm cultivations. Through fine-scale mapping and carbon accounting, we demonstrated that REDD+ can outcompete oil-palm in regions with low suitability, with low carbon prices and low carbon stock. In areas with medium oil-palm suitability, REDD+ could outcompete oil palm in areas with: very high carbon and lower carbon price; medium carbon price and average carbon stock; or, low carbon stock and high carbon price. Areas with high oil palm suitability, REDD+ could only outcompete with higher carbon price and higher carbon stock. In the coarse-scale model, oil-palm outcompeted REDD+ in all cases. For the fine-scale models at the landscape level, low carbon offset prices (US $3 MgCO_2_e) would enable REDD+ to outcompete oil-palm in 55% of the unprotected forests requiring US $27 million to secure these areas for 25 years. Higher carbon offset price (US $30 MgCO_2_e) would increase the competitiveness of REDD+ within the landscape but would still only capture between 69%-74% of the unprotected forest, requiring US $380–416 million in carbon financing. REDD+ has been identified as a strategy to mitigate climate change by many countries (including Malaysia). Although REDD+ in certain scenarios cannot outcompete oil palm, this research contributes to the global REDD+ debate by: highlighting REDD+ competitiveness in tropical floodplain landscapes; and, providing a robust approach for identifying and targeting limited REDD+ funds.

## Introduction

The international target of limiting global warming to 2°C or less cannot be achieved without tropical forest protection [1]. The carbon-based mechanism REDD+ (Reducing Emissions from forest Degradation and Deforestation), is aimed at mitigating climate change by providing an alternative solution to forest conversion to land uses such as oil palm [2]. To do this, REDD+ provides financial compensation from developed (Annex I) to developing (Annex II) countries who agree to decrease their deforestation and forest degradation rates by forgoing other land uses, thereby reducing their carbon dioxide emissions against a predicted reference level [3]. Concurrently, REDD+ aims to safeguard biodiversity through forest conservation and provide alternative income streams to landholders through REDD+ activities and/or direct payments for maintaining carbon stock aimed at poverty alleviation [4]. This seemingly win-win scenario was proposed in 2005 at the 11^th^ Conference of Parties (COP) of the United Nations Framework on Climate Change (UNFCCC), aimed at being a compliance-based mechanism with international regulations and standards [5]. Negotiations of REDD+ under the UNFCCC has quickly advanced with Annex I countries committed to mobilise US $ 100 billion by 2020 for a ‘green climate fund’ to facilitate REDD+ [6]. However, the global community is yet to establish successful negotiations and agreements on global climate change policies [7]. Until then compliance-based REDD+ is out of reach for the immediate future [8].

Despite such set-backs, a number of ‘REDD+ Readiness’ activities have been underway since 2007 (Conference of the Parties 13) to help prepare Annex II countries for REDD+ inclusion under the UNFCCC [9]. Such activities include national and/or sub-national strategy development, building of governance capacity; as well as, the implementation of pilot projects at local levels to understand how to synergise climate change mitigation with poverty and biodiversity loss alleviation [10]. For the time being however, REDD+ is limited to the voluntary markets where carbon is generally traded at a low price e.g. US $ 1–5 per metric ton (or Mega-gram) of carbon dioxide emissions (MgCO_2_e) [11]; although average annual carbon prices have been calculated higher at US $ 5.9 MgCO_2_e in 2011 [12], and US $ 7.8 MgCO_2_e in 2012 [12]. Whereas, estimates of carbon prices on a compliance market are generally assumed to be higher e.g. US $30 MgCO_2_e [13]. Low carbon pricing and largely unregulated standards for REDD+ projects, mobilised by voluntary market funds, have generated perceptions of inadequate overall financing to secure natural forested systems [9], especially those in regions of high value crops [14].

Deforestation can bring significant benefits to people through revenue from timber sales or agricultural livelihoods from conversion to pasture or crops [15]. By avoiding deforestation (and forest degradation) such benefits are forgone, known as ‘opportunity costs’ or the perceived forgone profit [16]. Understanding what the opportunity costs are for competing land uses, and how they vary across landscapes, is necessary for knowing benchmark values that need to be met by alternative finance mechanisms such as REDD+. Whether REDD+ can outcompete the opportunity costs of oil palm, especially on the voluntary market, is amongst one of the global debates happening in the REDD+ conversation [17–18]. That is because unlike other crops such as soya or maize, oil palm challenges the financial viability of REDD+ on three fronts. Firstly, oil palm can have high profit (higher than other large scale crops), driven by global demand coupled with high palm yield [19]. Secondly, as REDD+ is a carbon-based mechanism, its carbon accounting factors in carbon reference levels (i.e., carbon stock of proposed land use) which restricts carbon credits to net carbon saving only. Unlike low-lying, foliage based crops; oil palm can store fairly significant above-ground-carbon stock, impeding the net carbon saving from REDD+ [20]. Thirdly, in certain regions, for example in extensive areas of South East Asia, old growth or primary tropical forests are limited in extent with extensive forests having undergone varying degrees of logging thereby reducing their above-ground-carbon stock [21]. Within these contexts it begs the question as to whether REDD+ is a financially competitive alternative to oil palm cultivation in scenarios where forests are degraded and low carbon offset prices occur.

Several studies have assessed the financial viability of REDD+ with other land uses such as oil palm. These studies are based on various parameters including known carbon stock, opportunity costs, implementation costs, transaction costs (the various costs necessary for parties to have a transaction involving a REDD+ payment), and the accounting stance that may occur if a REDD+ project is implemented [15]. However, these studies have largely been based on coarse level average estimates for regions, or data from oil palm financial reports, hypothetical scenarios, and other surrogate information for these parameters. As a result the current body of literature is difficult to draw solid conclusions from with some analyses supporting the notion that carbon traded on the voluntary market can outcompete with oil palm [13, 17]. Whereas, other authors suggest that REDD+ can only be viable under the UNFCCC (i.e., on the compliance based market) [14]. Conflicting conclusions may impede the progress of REDD+ at many levels (e.g. international or national policy level, investor or landholder levels). Nuanced financial analyses are needed that reflect actual landscapes, as well as the policies that underpin them. Such analyses are fundamental to ensuring the economic viability of implementing REDD+ within a landscape. This is especially important in regions regarded as having potentially high opportunity costs like with oil palm [3]; as well as the feasibility for landholders to uptake REDD+ in the future.

This research contributes to a gap in the REDD+ literature by undertaking—to our knowledge—the first fine-scale (using regionally specific data) financial assessment of REDD+ (compliance and voluntary market) within a floodplain case study. A floodplain system was selected as these regions are some of the most biologically diverse terrestrial systems globally, yet are highly threatened by the expansion of oil palm agriculture [22]. Understanding where and to what degree REDD+ might be financially competitive with oil palm could therefore help target limited REDD+ funds to key biodiversity areas such as floodplains.

Specifically in this paper, we assess whether REDD+ could provide a financial solution for landholders (both large commercial plantations and smallholders) to forgo converting unprotected forest to oil palm in the Kinabatangan floodplain in Sabah, Malaysian Borneo. We generated fine-scale categorical forest carbon stock maps in metric tons (or Mega-gram) of carbon (MgC), using field data and an Object-Based Image Analysis (OBIA) approach and best available data for oil palm carbon stock for the region, to generate the REDD+ reference level (i.e., oil palm carbon stock). We undertook fine-scale carbon accounting for REDD+ using different carbon price scenarios to quantify net present value (NPV) of degraded yet biologically valuable unprotected forests and compared these with potential NPV of variable oil palm productivity calculated for the study region [23]. Additionally, we undertook coarse-scale economic models to compare results against those generated in the fine-scale models. We also considered policy constraints that limit landholders to enter into a REDD+ program and discuss the issues regarding current policy for the case study region.

## Materials and Methods

### Ethics statement

To conduct the research in Sabah, Malaysia, a research permit was obtained initially from the Economic Planning Unit (EPU) then from the Sabah Biodiversity Centre (SaBC). Additionally, the Sabah Wildlife Department was the official in-country counterpart for this research and Sabah Forestry Department was a partner to this study.

### REDD+ context

Malaysia is a ratified party of the Kyoto Protocol under the UNFCCC and has committed to reduce National Level Carbon emissions by 40% by 2020 referenced against 2005 levels [24]. Malaysia has identified REDD+ as one strategy to achieve this target, and has undertaken ‘REDD+ Readiness’ to develop reference levels, strengthen governance capacity, source financing etc, at national and State levels [24]. Additionally, Sabah is a recipient of a sub-national/State EU-REDD funded project. This EU-REDD project is providing technical support for monitoring, reporting and verification (MRV), safeguard development, and enhancing capacity; as well as implementing three, four year, pilot studies to assess local community and biodiversity aspects of REDD+, with one located in the Lower Kinabatangan floodplain. Despite multiple REDD+ activities in Sabah, to our knowledge no financial assessments have been undertaken to assess the economic viability of this mechanism. Ensuring the economic feasibly of REDD+ in potentially high opportunity cost landscapes is vital to ensure its effectiveness [3].

### Study area

Our study largely focused on the unprotected forests left (30,173 ha) within the Lower Kinabatangan floodplain study region, which encompassed a total area of 520,269 ha and comprised of 48% (250,617 ha) of cultivated oil palm, and 48% of protected, managed or unprotected forest (as of 2010–11) [23]. Managed forests include Commercial Forest Reserves and Mangrove Forest Reserves, with fully protected areas including the Lower Kinabatangan Wildlife Sanctuary and Virgin Forest Reserves, all herein known as ‘protected areas’. Specific forest types in this region, are associated with mangrove, flooded forest, and dry (humid) forest systems (see Table 1 for details), many of which are considered threatened [25]. Most forest in the region has undergone mechanised intensive selective logging, and significant forest loss has occurred resulting in severe forest fragmentation [26–27]. Although degraded and fragmented, remaining forest has significant carbon stock [20]. Moreover, unprotected forests are crucial in maintaining connectivity between protected areas and in providing habitat for significant populations of endemic and threatened IUCN Red List mammal species e.g. the Bornean orangutan (*Pongo pygmaeus morio*) circa 800 individuals [28]; proboscis monkey (*Nasalis larvatus*) circa 1,450 individuals [29–30]; and the Bornean elephant (*Elephas maximus borneensis*) circa 300 individuals [31].

**Table 1:** Forest systems and forest types with corresponding extent, plot data extent (ha), estimated annual flooding periods, and predicted oil palm productivity class.

Forest type	Plot data (ha)	Annual flooding period	Oil palm suitability classes (potential)	Total extent (ha)
**Mangrove System**				
Beach forest	1	Tidal	Underproductive at ≤25%	655
Mangrove forest	1	Tidal	Underproductive at ≤25%	506
Nipah palm forest	-	Tidal	Underproductive at ≤25%	1,219
Transitional forest	1	Semi-tidal	Underproductive at ≤25%	3,282
**Seasonally Flooded Forest System**				
Freshwater swamp forest	9	>6mths	Underproductive at ≤25%	5,563
Seasonal freshwater swamp forest	21	3-6mths	Underproductive at ≤25%	4,248
Peat swamp forest	6	>6mths	Underproductive at ≤25%	30
Swamp	-	>9mths	Underproductive at ≤25%	702
**Humid Forest System**				
Lowland dry forest	40	Rarely/ <3mths	Full stand	8,315
Lowland dry dipterocarp forest	18	Never/Rarely	Full stand	1,012
Limestone Forest	6	Never/Rarely	Underproductive at ≤25%	287
**Mixed Forest Systems**				
Degraded (Mixed habitats)	7	Unknown	Underproductive at 50%	4,352
Total number of hectares	110			30,173

Despite the conservation value of these unprotected forests, more than 64% have been allocated (otherwise known as ‘alienated’) by title for conversion to either large commercial oil palm plantations (under Country Land Title) or oil palm smallholdings (under Native Title) [23]. What little land is untitled is classified as State land [23]. These data are from undated cadastral maps and therefore the actual percentage of alienated land is likely higher than 64%. This means that most of the unprotected forests are likely threatened with conversion in the imminent future. However, floodplains are heterogeneous in their suitability for oil palm cultivation and in the study region it is predicted that at least 54% of the unprotected forest is unsuitable for this crop, due to tidal or seasonal monsoon inundation and an inability for palms to tolerate such flooding [23]. If converted, costs of oil palm establishment would likely outweigh potential revenue.

For the existing oil palm landscape, a concurrent study has mapped the suitability and profitability of oil palm cultivation in the Kinabatangan. Areas that were regarded as ‘Full stand’ (i.e., young palm between 3–6 years, mature palm between 7–24 years that ranged between 76%-100% palm capacity, cleared areas, and new plantings of less than 2 years) had an estimated annual Net Present Value (NPV) of US $413-$637 per hectare over 25 years (ha^-yr^). Areas that were underproductive were classified as: ‘Underproductive at 75%’ which assumed 51%-75% palm capacity; ‘Underproductive at 50%’ with 26–50% palm capacity with an estimated NPV of US $-55-$169/ha^-yr^; and ‘Underproductive at ≤25%’ that assumed 0–25% palm capacity and NPV of US $299-$-65 ha^-yr^ [23]. The underproductive classes in total encompassed 20% of the oil palm extent (or 51,466 ha); with the ‘Underproductive at ≤25%’ totalling 15,810 ha [23]. Understanding the heterogeneous nature of floodplains for palm oil production is critical when understanding where REDD+ may outcompete potential high profit crops like oil palm.

### Forest above-ground carbon biomass

Many ecosystem services are provided by lowland forests such as: sequestering and storing carbon [21], mitigating river bank erosion, regulating flood events, regulating local temperatures/climate (as well as mitigating global climate change) [32–33], prevention of certain diseases (e.g. flooding related malaria) [34–35], providing spawning grounds for local fisheries and buffering against sea level rise [36]. Additionally, some forests provide cultural benefits [37–38], and forest products for peoples livelihoods [35]. For this study, above-ground-carbon storage was solely focused on to explore carbon-based conservation opportunities under REDD+ which is gaining momentum in Sabah. One caveat to the carbon models described below was the exclusion of below-ground-carbon. Although below-ground-carbon can be very significant, especially in mangrove and flooded forests [39], no regional data were available and use of surrogate data would have introduced unknown degrees of uncertainty into the analyses [40]. A conservative REDD+ model therefore was developed for above-ground-carbon only.

Data were collected from 230 plots covering 110 ha for ten of the forest types identified in the floodplain (Table 1). To develop the carbon maps, 110 hectares (230 plots) of forest mensuration data were used as training and testing data for the carbon classification. Of this, 72 hectares were derived from Sabah Forestry Department’s (SFD) 2006/2007 forest inventory data that used transect line surveys of 20 m wide and ranging from 750 m to 2 km in length; and 8.4 hectares from permanent plots collect between July-October 2009. Nine hectares of supplementary data from two Forest Reserves (collected by author AM using strip transects, in April and November 2008). Seven hectares were collected by author NKA using plot and strips transects in December 2010 and January 2011; with the remaining plot data provided by authors MA, IL and BG using 0.25 ha permanent plots (data collected in 2010/2011).

Measurements in these plots included: Diameter at Breast Height (DBH) for trees ≥10 cm; species or genus vernacular/scientific names; habitat type; and degradation level. To derive carbon estimates, species or genus wood density averages were used from Brown [41] and the World Agro-forestry Centre Wood Density Database (www.whrc.org). If values were unknown a standard value of 0.5g cm^-3^ was used to ensure estimates were conservative [42]. Allometric calculations were considered, needed to convert plot data to carbon stock values i.e., Above-Ground Biomass (AGB). However, due to limitations inherent in the existing data, most plots had species level and DBH information only and not height. As a result, the allometric calculation by Chave et al. [42] was used, which based AGB estimates on DBH and wood density values for tropical forests—deemed appropriate by previous studies for the region [20]. Using an allometric calculation that omits height does add error into the results [20]; and we discuss potential implications of this in the discussion. However, as plot level data was later converted to broad carbon stock classes, it is likely that any errors would have minimal impact on the overall carbon mapping results. The allometric calculation was as follows: where *D* refers to DBH (in cm), *ρ* refers to wood density (g cm^-2^):

$$\text{AGBest}=p\text{ x exp}\left(-1.499+2.148\text{ xln }\left(D\right)+0.207\text{ x }{\left(\text{ln}\left(\text{D}\right)\right)}^{2}-0.02081\;{\left(\text{ln}\left(\text{D}\right)\right)}^{3}\right)$$

#### Forest carbon mapping

A step-wise Object-Based Image Analysis (OBIA in eCognition Developer 8.7) was employed for the carbon classification. OBIA was used as it creates meaningful “objects” by segmenting images into groups of pixels based on similar spectral signatures and other characteristics e.g. textural information [43–44]. Additionally, this methods was selected to be most appropriate as it omits the salt-and-peppering issues often found in pixel based methods, for an example of this within the study region see Morel et al. [20], compared to an OBIA approach [23]. OBIA permits multiple satellite images to be used within the two carbon classifications (one in the eastern region and one in the western region).

Satellite imagery used included SPOT5 10 m satellite imagery, one captured on 25/11/2007 and two on 19/06/2009 from Planet Action (www.planetaction.org), and two Landsat TM 30 m imagery captured on 27/07/2006 and 11/08/2009 downloaded from www.usgs.gov. These images were the most recent images available, with least cloud cover for the region. Importantly, the 2007 SPOT5 data and 2006 Landsat TM data were used in the eastern region’s classification, and the 2009 SPOT5 and 2009 Landsat TM images for the western regions classification. Seasonality within the two classifications was not regarded as an issue, as all images were captured within the dry season. All satellite images were orthorectified and indices calculated for: Normalized Difference Vegetation Index (NDVI); Normalized Difference Moisture Index (NDMI); and Soil Adjusted Vegetation Index (SAVI). Spectral transformations were performed including reflectance for SPOT5 and Tasseled Cap transformation on the Landsat TM5 Images [45]. Additionally, two SPOT5 2.5 m images, from Planet Action captured on the 12/12/2010 (eastern region) and 01/06/2011 (western region) were used to produce a generic forest extent auxiliary layer, digitised using ArcGIS 10, to restrict the classifications to areas of known forest.

The 230 plots were split into halves, one used for training the model and the second for testing the result. On each plot a carbon class was assigned based on the content of carbon per ha, using metric tons of carbon (MgC). Classes were: Class 1 = ‘<50 MgC/ha’; Class 2 = ‘50–100 MgC/ha’; Class 3 = ‘100–200 MgC/ha’; Class 4 = ‘200–300 MgC/ha’; Class 5 = ‘300–400 MgC/ha’; Class 6 = ‘>400 MgC/ha’. No data for nipah palm forest or swamp was available. Instead for both nipah palm and swamp ‘objects’ were manually selected within eCognition and classified as the lowest carbon class (i.e., Class 1 = <50 MgC/ha). This category was assigned, as no data were found in peer-review or grey literature on carbon stock. Moreover, nipah palm is a frond based plant; and, swamp areas often lacked trees and consisted of reed-like vegetation, therefore we assumed little above-ground carbon. It is noted that these two vegetation types may have high soil carbon values [46], but soil carbon was not included in this study.

For image segmentation and object generation, SPOT5 10 m data were employed to delineate ‘objects’. Scale parameters were trialled, and a scale of 15 was used, deemed most suitable through visual inspection and ground knowledge. Colour/shape and smoothness/compactness were set at 0.9/0.1 and 0.5/0.5 respectively [47]. The 2010/2011 generic forest extent layer was used to define the classification area; and used all spatial data within the rule set development and identification of the features with the highest discrimination ability. A Classification and Regression Tree analysis (CART) algorithm was used as it performed better than other trialled classifying algorithms (Support Vector Machine and Byes) in reproducing the training set.

The carbon classifications were exported as shapefiles and ‘merged’ to form one continuous layer. To quantify landscape carbon stock the mid-point for each carbon class was used (i.e., Class 1 = 25 MgC/ha; Class 2 = 75 MgC/ha; Class 3 = 150 MgC/ha; Class 4 = 250 MgC/ha; Class 5 = 350 MgC/ha; Class 6 = a conservative 450 MgC/ha) and multiplied by the number of hectares per class. A classification accuracy assessment was performed using an error matrix on the second half of the plots at object-level and overall accuracy and kappa statistics were calculated [47]. Cadastral data from Abram et al. [23] were used to inform title information for the unprotected forest and to calculate carbon stocks allocated for conversion to oil palm.

### Oil palm above-ground carbon biomass

**Fig 1:**
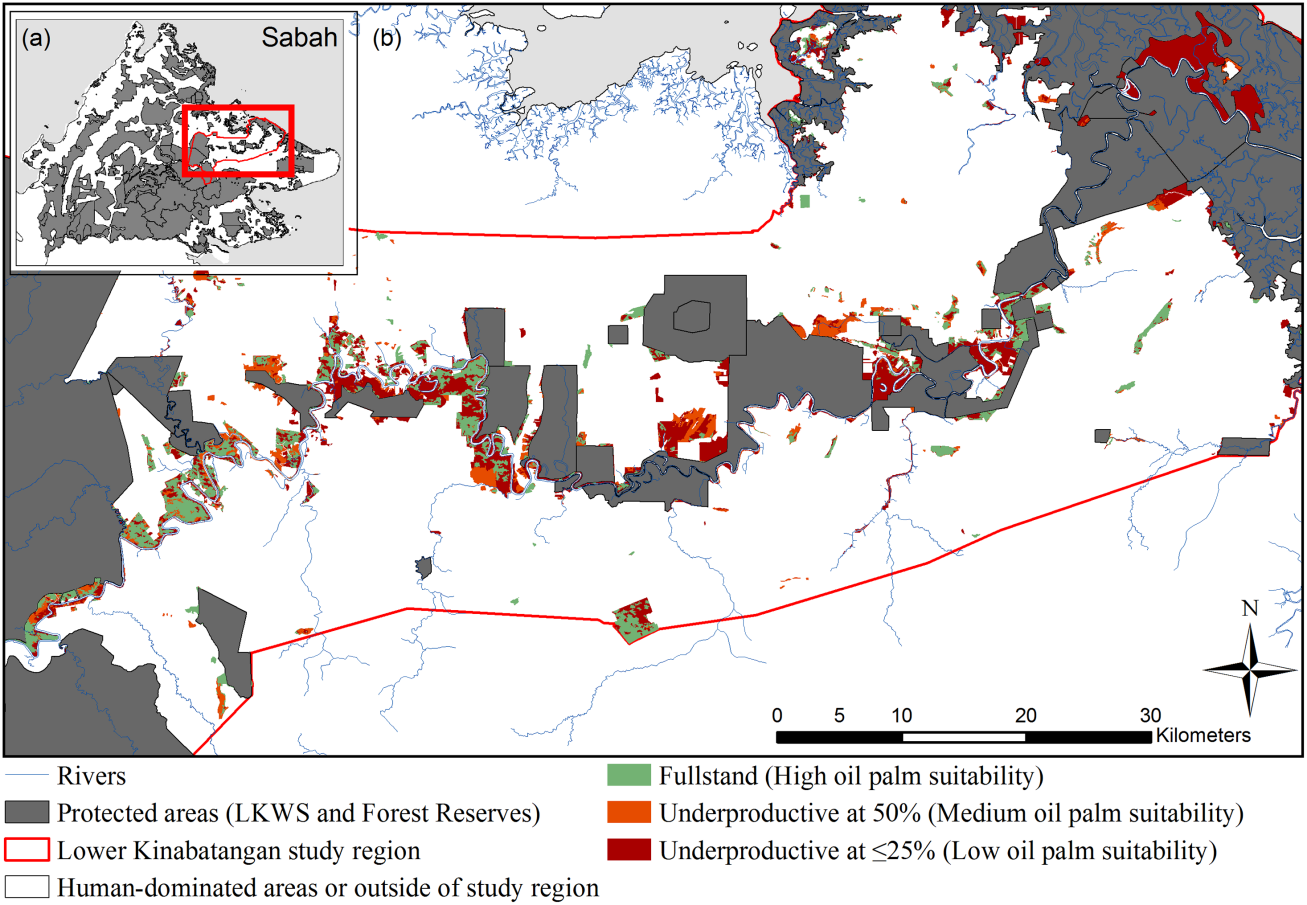
Map of predicted oil palm productivity classes in unprotected forest in Sabah context. (A) Map of study region in Sabah (red outline) in Malaysian Borneo. (B) Map showing the areas of unprotected forest reclassified to potential oil palm productivity classes of Full stand totalling 9,327 ha (green), Underproductive at 50% at 4,352 ha (orange), and Underproductive at ≤25% totalling 16,492 ha (red), within the study region that is dominated by human land use mainly oil palm (white). The protected area network (grey) includes fully protected forests along with use Forest Reserves (use forest).

Oil palm above-ground carbon biomass was estimated as the reference level for the REDD+ models. We used above-ground carbon biomass oil palm data collected within the region, for methodological details see Morel et al. [20]. Data composed 22 plots of 0.25 ha, in 11 age categories (in years, including years 2–5, 7–8, 13, 15–16 and 18–19). For those categories that lacked data we: (1) calculated the mean value of the missing year, using data from the previous and following years; or, (2) if consecutive years were missing we estimated the incremental value per year. Furthermore, the oldest value we had was for palm at 19 years of age. As our economic models were for a 25 year crop life, we held the 19 year carbon biomass value for years 20 to 25, which likely overestimated the actual carbon value as palms. From these data, carbon estimates were calculated at a palm level for each age category (in MgC/ha), and a time-averaged carbon estimate was calculated over a 25 year crop lifespan. Average palm carbon was then multiplied by the number of palms per oil palm suitability class, predicted for the region see Abram et al. [23] and see Fig 1. These classes used the maximum number of palms in that class in order to estimate the upper range carbon reference levels for the oil palm classes. These classes included: (1) ‘Full stand’ areas which in this study represented regions with 136 palms per hectare or 100% palm capacity; (2) ‘Underproductive at 50%’ which assumed 68 palms per hectare or 50% palm capacity; and (3) ‘Underproductive at ≤25%’ that assumed 25% palm capacity or 34 palms per hectare.

### Economic modelling

#### Fine-scale REDD+ economic modelling

Net present value (NPV) of four REDD+ price scenarios were calculated. Three voluntary market price scenarios were used: low (US $3 MgCO_2_e); middle (US $7.8 MgCO_2_e) as a bilateral agreement price for 2012 [48]; high (US $15 MgCO_2_e); and a higher value of US $30 MgCO_2_e, based on a full compliance mechanism under the UNFCCC. For the accounting stance, two approaches were used. Firstly, an upfront payment scheme was employed which assumed all carbon (forest) was simultaneously incorporated into a REDD+ project in year one; with a constant carbon price used and paid upfront in the first year. This scheme assumed a bilateral voluntary market or compliance agreement which may be plausible, and because large up-front payments have been seen to be favoured by farmers for REDD+ initiatives (p. 128 [49]); though obtaining contractual agreements from landholders in one year may be tenuous. The second accounting stance was more conservative and assumed a 50% payment in year one, with remaining payments using a constant carbon price made in each subsequent year.

Model timeframes spanned 25 years, allowing direct comparisons with oil palm economic models. For REDD+ implementing costs, we used values from Butler et al. [17] at: US $25/ha for year one’s establishment costs, calculated at US $754,325; and US $10/ha for running costs in each subsequent contract year, amounting to US $ 301,730 annually. We justify use of these values as when compared with the regions on-going EU-REDD+ pilot study, these annual costs are marginally higher (i.e., the EU-REDD+ project has an annual budget of US $296,000), and has no additional establishment budget. Although, the current EU-REDD+ pilot study and any future REDD+ project will differ in their objectives, and consequently will have differing associated budgets, the Butler et al. (2009) values seem to be reasonable for this region. We used a discount rate of 11% per annum, as used by industry in the region in 2011 [23]; as well as at 5%, 8% and 14% to test model sensitivity to this variable.

For the carbon values, we used a mid-point of MgC/ha within each carbon class, minus the reference level carbon (i.e., the estimated upper range value of MgC/ha for each oil palm suitability class), and converted net carbon values to metric ton of carbon dioxide emissions equivalent per hectare (MgCO_2_e/ha), by multiplying by the molecular conversion factor of 3.67 [1]. The MgCO_2_e/ha was then used to calculate the NPV for each REDD+ carbon offset price. See Tables A-F in S1 File for input values in these models.

#### Fine-scale oil palm economic modelling

To understand benchmark opportunity costs, existing economic models were slightly modified for three oil palm classes (identified in a concurrent study, see Table 1, Fig 1) that will likely result if the unprotected forest is converted to oil palm [23]. These included areas predicted to be: Full stand, Underproductive at 50% and Underproductive at ≤25% classes. Much of the data (yields, costs, price) used in the economic models were based on summary data of actual 2011 values from plantations, these data of which were collated by a land valuation agency (C H Williams Talhar & Wong) for use in their land valuations.

Yield curves for these classes were developed by Abram et al. [23] that a 25 year crop life, assumed 136 palms per hectare (upper range of the Full stand areas) per industry standard [50], and actual yield data of 30 metric tons (t) of fresh fruit bunches (FFB) yield per hectare (ha) in peak years (years 8–11) declining to 17 tFFB/ha in year 25, averaging at 21.92 tFFB^-yr^. A direct relationship was assumed between the number of palms per hectare and yield. As a result, annual yields for the other models were generated by calculating the proportion of yield (in tFFB/ha) for the proportion against the ‘Full stand’ yield values. Only upper yield ranges in each class were used in this study, to try and achieve as robust conclusions from these models in regards to REDD+ viability compared with oil palm. Meaning, for ‘Full stand’ we assumed 136 palms per ha (i.e., an average yield of 21.92 tFFB/ha^-yr^), for ‘Underproductive at 50%’ we used 50% palm capacity equivalent (averaging 10.96 tFFB/ha^-yr^), and for ‘Underproductive at 25%’, 25% palm capacity per hectare (averaging 5.48 tFFB/ha^-yr^). Fresh fruit bunches were used as the revenue unit instead of crude palm oil (CPO) as for smallholders and commercial estates with no processing mills, revenue is derived from the sale of FFB.

A constant FFB price of US $178 tFFB was used (based on 2011 averages from the east coast of Sabah). Costs included: General charges (or Joint Estate costs); Field upkeep (weeding; manuring; pruning; pests and disease treatment; supplying; infrastructure etc); and Harvesting and transport. Costs were kept constant in the four models except for ‘supplying’ which in commercial estates is undertaken within the first two years of production to ensure homogeneous age blocks [51]. Although the cost of supplying is small, these were altered according to how many palms were assumed to have died. Cost data were calculated from estates past actual costs, their budgeted future costs and typical industry average costs for 2011 (obtained from C H Williams Talhar & Wong). These costs were cross referenced and supplemented with state wide data on costs from 2008 [51]. A commercial estates approach (and not a smallholders approach) was used due to data availability and use in translating industry standards into the economic models. To amend these models to reflect ‘new plantings’ of oil palm, we included non-recurrent costs of felling of trees and ground preparation calculated at US $ 1214.8/ha in year one [51]. An annual discount rate of 11% was used to reflect that used by industry in Sabah in 2011, and 5%, 8% and 14% discount rates were also used to test model sensitivity (see Tables A-C in S2 File, for details).

#### Fine-scale versus course-scale economic modelling

To demonstrate the inherent issues with using course or average values when modelling opportunity costs in landscapes, we also modelled REDD+ using both accounting stances already outlined with the same carbon price values, and a discount rate of 11%. We use our averaged above-ground-carbon value developed in this study for forested areas (see results section). For oil palm above-ground-carbon we used 52 MgC/ha, calculated for mature oil palm in the same region by Morel et al. [20]. For the oil palm opportunity costs, we use the values from our ‘Full stand’ oil palm model which averages at 21.42 tFFB/ha, which is marginally lower than Sabah’s 2008 average (22.65 tFFB/ha) [51].

#### Spatial patterns of profit

We spatially allocated where REDD+ NPV was higher than oil palm, across the unprotected forest landscape, to help inform potential future decisions on where to implement REDD+, for each carbon price. This was done by integrating the spatial and economic datasets using ArcGIS 10.1. We also enumerated MgC and MgCO_2_e (based on mid-point carbon class values) of forest carbon that would be safeguarded if REDD+ was implemented for both payment schemes; and estimated the total funds needed for the 25 year REDD+ project for those areas.

## Results

### Carbon stock

Overall classification accuracy was 86.9%, with a kappa statistic of 0.82, indicating good performance [52], using an error matrix method and 115 testing plots. For the, description of possible classification errors, classification error matrix and the carbon map see S3 File (Table A and Figure A in S3 File). A total of 50.21 million MgC was estimated for the entire forested area of the floodplain (Table 2). Of this, unprotected forest comprised 9.4% (4.7 million MgC) of total above ground forest carbon (Table 2); with an average value of 156 MgC/ha. Of carbon stored outside protected areas, 63% (2.9 million MgC) was allocated for agriculture (but had not been removed as of 2010/11); and 12% (0.59 million MgC) occurred within demarcated (assumed under application) State Land, and 25% (1.1 million MgC) on non-demarcated State Land (Table 3).

**Table 2:** Carbon classes with mean carbon value (metric tons of carbon per ha) per class used to calculate carbon stock (MgC); total forest and unprotected forest extents (ha) with predicted carbon stock (MgC).

Carbon Class	Mean MgC/ha	Extent of all forest (ha)	Extent of unprotected forest (ha)	Total MgC in all forest	Total MgC in unprotected forest
Class1_<50	25	26,000	4,249	650,000	106,225
Class2_50–100	75	26,308	3,989	1,973,100	299,175
Class3_100–200	150	91,012	15,387	13,651,800	2,308,050
Class4_200–300	250	53,621	3,851	13,405,250	962,750
Class5_300–400	350	40,103	1,841	14,036,050	644,350
Class6_>400	450	14,472	856	6,512,400	385,200
Total	-	251,516	30,173	50,228,600	4,705,750

**Table 3:** Carbon stock (MgC) in unprotected forests that are alienated (allocated under Native Title or Country Land Title); or in State land that is demarcated (assumed to be under application and may now be alienated) or un-demarcated.

Land title types	Total MgC in unprotected forest
Native Title	1,376,453
Country Land Title	1,602,923
State Land (demarcated)	590,908
State Land (un-demarcated)	1,135,618
Total MgC	4,705,900

Our carbon reference level estimates for potential oil palm, based on a 25 year life span, for three suitability classes were calculated at: (1) Full stand equalled 46 MgC/ha; Underproductive at 50% at 20 MgC/ha; and, 7 MgC/ha for the Underproductive at ≤25%.

### Oil palm NPV potential in unprotected forest

Calculated averaged annual NPV of new plantings for oil palm, using a discount rate of 11% for the three suitability classes were as follows: Full stand areas US $594 (and US $1,206, US $836, and US $429 for the discount rates of 5%, 8%, and 14% respectively); Areas Underproductive at 50% US $129 (and US $327, US $205, and US $72 for the discount rates of 5%, 8%, and 14% respectively); and, Underproductive at ≤25%—US $109 (and—US $112,—US $110, and—US $107 for the discount rates of 5%, 8%, and 14% respectively) (see Tables A-C in S2 File for calculations).

### REDD+ versus oil palm NPV in unprotected forest

**Fig 2:**
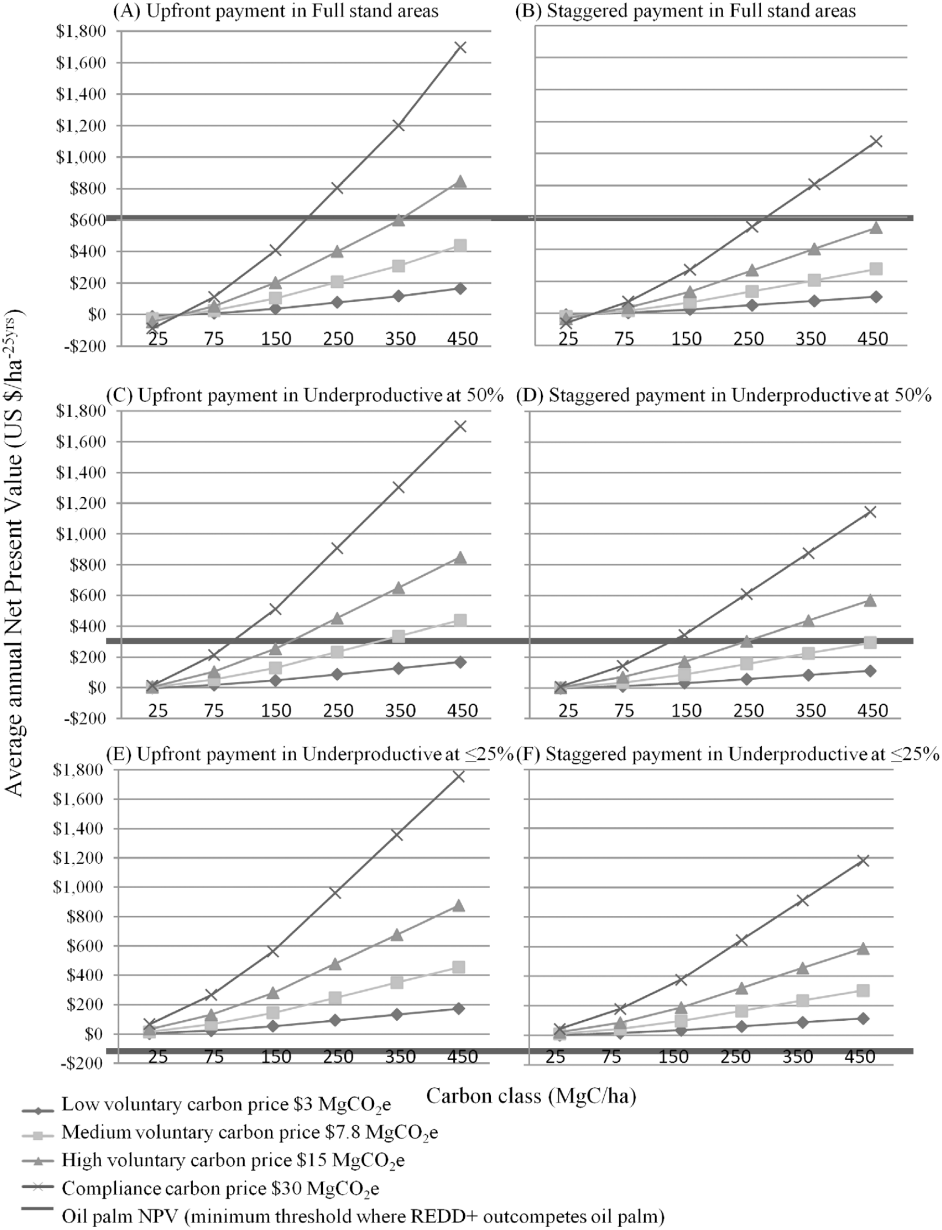
Predicted annual net present values of REDD+ economic models (in US $ per ha) within three carbon price scenarios of variable oil palm suitability in the region. Figures show the averaged annual Net Present Values of REDD+ models (over 25 years) for four carbon prices (US $3 MgCO_2_e, US $7.8 MgCO_2_e, US $15 MgCO_2_e, and US $30 MgCO_2_e) based on hectare level above-ground-carbon stock classes (MgC/ha) for a: (A) Upfront REDD+ payment in areas predicted to have full stand oil palm: (B) Staggered Payment in areas predicted to have full stand oil palm; (C) Upfront REDD+ payment in areas predicted to be underproductive at 50%; (D) Staggered REDD+ payment in areas predicted to be underproductive at 50%; (E) Upfront REDD+ payment in areas predicted to be underproductive at ≤25%; (F) Staggered REDD+ payment in areas predicted to be underproductive at ≤25%. The thick dark grey line shows the estimated NPV of oil palm within that suitability class, below this line oil palm is more competitive and above this line shows the scenarios in which REDD+ is financially more competitive.

Averaged annual NPV’s for REDD+ outcompeted those of new oil palm plantings within certain contexts in both the upfront and staggered payment schemes (Fig 2, and Tables A- F in S1 File). However, in most cases the upfront payment scheme had higher NPV than that of the staggered payment scheme. Per suitability class, NPV models under the staggered payment accounting stance ranged from 63% (with a 14% discount rate) to 78% (with a 5% discount rate) that of the NPV calculated for the upfront accounting stance with the model with the 11% discount rate being 66% that of the alternative payment scheme (see Tables A- F in S1 File; and, Table H in S1 File for the sensitivity analyses).

#### Areas of high oil palm suitability

In areas of high oil palm suitability (i.e., Full stand), an upfront REDD+ payment scheme outcompeted oil palm when: (1) the carbon price was US $15 MgCO_2_e in areas with ≥350 MgC/ha; or, (2) carbon price was US $30 MgCO_2_e in areas with ≥250 MgC/ha (Fig 2). Negative NPV for REDD+ occurred for all carbon price scenarios in the 25 MgC/ha carbon class. The 14% discount rate models mirrored the above, whereas the REDD+ models with 5% or 8% discount rate outcompeted oil palm when the carbon price was US $30 MgCO_2_e in areas with ≥350 MgC/ha.

For the staggered payment scheme that used a discount rate of 11%, REDD+ outcompeted oil palm only when the carbon price was US $30 MgCO_2_e in areas with ≥350 MgC/ha. This was mirrored for the 8% discount rate models (Fig 2). Using a 5% discount rate however, meant that only the carbon price of US $30 MgCO_2_e in areas with ≥450 MgC/ha was more competitive under REDD+. For the models with 14% discount rate, REDD+ was more competitive than oil palm when there was a carbon price of US $15 MgCO_2_e in areas with ≥450 MgC/ha, or a price of US $30 MgCO_2_e in areas with ≥250 MgC/ha.

#### Areas with medium oil palm suitability

For the 11% discount rate models in areas of medium oil palm suitability (i.e., Underproductive at 50%), the upfront REDD+ payment scheme outcompeted oil palm when: (1) the carbon price was US $3 MgCO_2_e in areas with ≥350 MgC/ha; (2) the price was US $7.8 MgCO_2_e and US $15 MgCO_2_e in areas with ≥150 MgC/ha; and (3) the carbon was priced at US $30 MgCO_2_e with ≥75 MgC/ha (Fig 2). Marginal negative REDD+ NPV occurred for the combinations of the lowest carbon price and lowest carbon class. Under a carbon price of US $3 MgCO_2_e models with either 5% or 8% discount rate, oil palm outcompeted REDD+ across all carbon classes. However under the 8% discount rate models REDD+ could still outcompete oil palm where there is a carbon price of US $7.8 MgCO_2_e in areas with ≥250 MgC/ha; US $15 MgCO_2_e in areas with ≥150 MgC/ha, and US $30 MgCO_2_e with ≥75 MgC/ha. The 14% discount rate models increased REDD+ competitiveness.

Under the staggered payment scheme using an 11% discount rate, REDD+ outcompeted oil palm when: (1) the price was US $7.8 MgCO_2_e in areas with ≥350 MgC/ha; (2) the carbon was priced at US $15 MgCO_2_e in areas with ≥15 MgC/ha; and, in areas with the carbon price at US $30 MgCO_2_e in areas of ≥75 MgC/ha (Fig 2). Decreasing the discount rate similarly decreased the competitiveness of REDD+ however under the 8% discount rate scenarios, REDD+ still outcompeted oil palm in the same carbon classes for the carbon price of US $7.8 MgCO_2_e, in areas with ≥250 MgC/ha and US $15 MgCO_2_e, and in areas of ≥150 MgC/ha for the compliance based carbon price. Again, the 14% discount rate models increased REDD+ competitiveness.

#### Areas with low oil palm suitability

In areas with low oil palm suitability (i.e., Underproductive at ≤25%), all carbon price scenarios (under all discount rates) in both the upfront and staggered payment schemes had equal or higher (positive) NPV under REDD+ than oil palm (Fig 2).

#### Fine-scale economic modelling values

For the upfront payment scheme REDD+ NPV’s were calculated at: US $37, US $103, US $202, and US $409, for the carbon prices of US $3 MgCO_2_e, US $7.8 MgCO_2_e, US $15 MgCO_2_e, and US $30 MgCO_2_e, respectively. For the staggered payment scheme the REDD+ NPV’s were US $24, US $68, US $135, and US $274, for US $3 MgCO_2_e, US $7.8 MgCO_2_e, US $15 MgCO_2_e, and US $30 MgCO_2_e, respectively. Under our course-scale economic modelling REDD+ was not competitive with oil palm, which had an average annual NPV of US $594/ha^-yr^yrs.

#### Spatial patterns of opportunity cost

**Fig 3:**
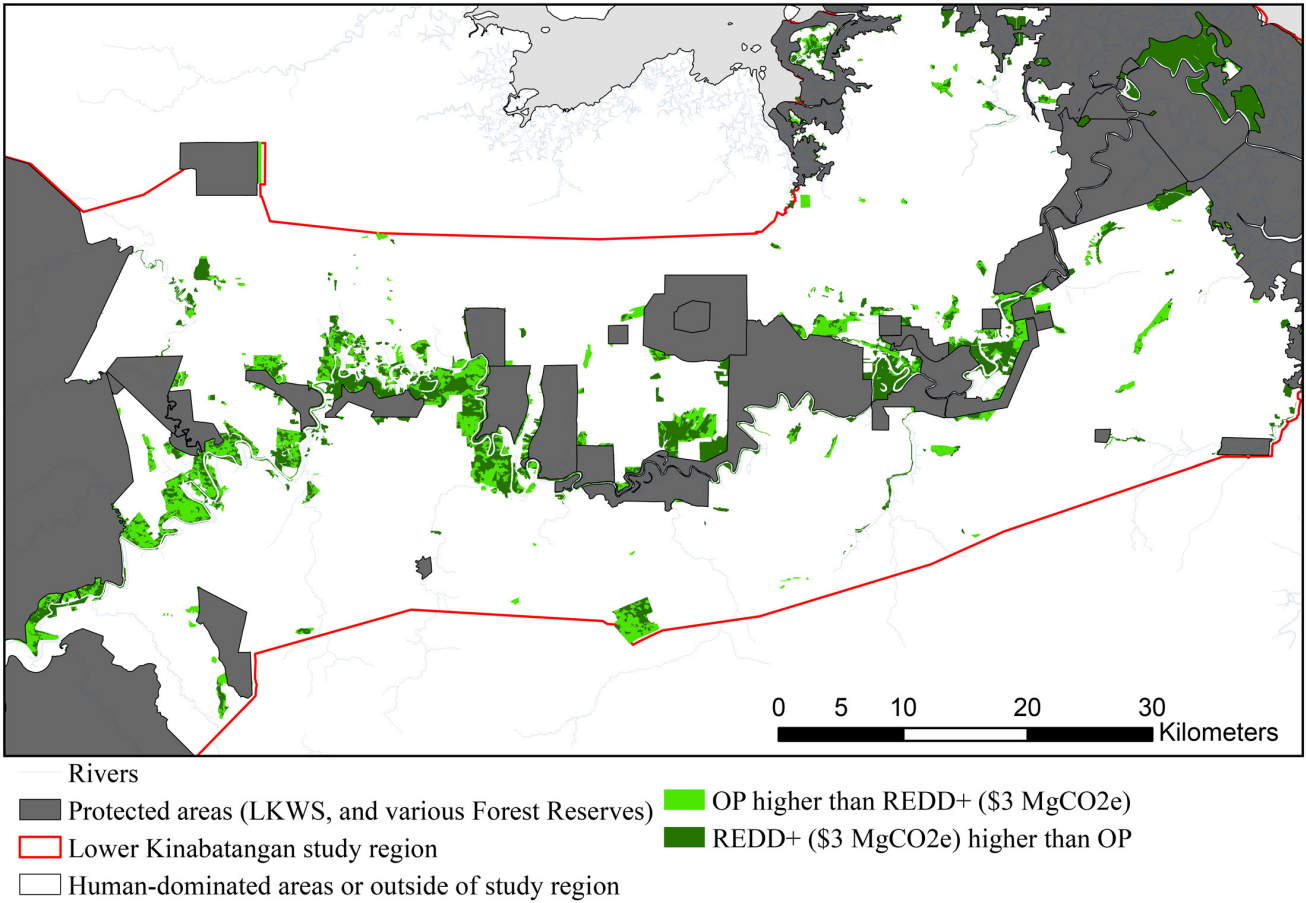
Spatial allocation of the highest NPV under REDD+ or oil palm. Spatial allocation of highest opportunity cost (Net Present Value) within the unprotected forests, between business-as-usual (BAU) conversion to oil palm (O.P.) verses with REDD+ project with a low carbon price (US $3 MgCO_2_e) and an upfront payment accounting stance.

REDD+ outcompeted oil palm in both accounting stances across 55% of the unprotected forest, on the low voluntary carbon price (US $3 MgCO_2_e) requiring around US $27 million to safeguard these forests for 25 years (see Fig 3 and Table 4). For the highest carbon price (US $30 MgCO_2_e) REDD+ outcompeted oil palm across 74% under the upfront payment accounting stance (this was the maximum extent) requiring a significant US $416 million, see Table 4 for these and all other scenarios.

**Table 4:** Outputs from the four carbon offset price scenarios. Table shows the extent (in ha and %) of the unprotected forest that would derive higher NPV under a REDD+ project than oil palm, the total tons of metric carbon stock (MgC) and carbon dioxide emissions equivalent (MgCO_2_e) secured in these areas, along with total carbon offset funds needed (US $) to secure these areas over 25 years.

Carbon price (US $/ MgCO_2_e)	Extent to which REDD+ outcompetes oil palm in unprotected forest in ha (and %)	Total MgC (summation of carbon within the extent where REDD+ outcompetes oil palm)	Total MgCO_2_e (MgC multiplied by 3.67)	Total REDD+ funds needed (MgCO_2_e multiplied by the carbon price in US $)
**Full payment scheme**				
Voluntary low case US $3 MgCO_2_e	16,629 (55)	2,495,980	9,160,247	$27,480,740
Voluntary mid case US $7.8 MgCO_2_e	18,999 (63)	2,887,380	10,596,685	$82,654,140
Voluntary high case US $15 MgCO_2_e	20,376 (68)	3,413,530	12,527,655	$187,914,827
Full compliance US $30 MgCO_2_e	22,178 (74)	3,779,505	13,870,783	$416,123,501
**Staggered payment scheme**				
Voluntary low case US $3 MgCO_2_e	16,546 (55)	2,461,415	9,033,393	$27,100,179
Voluntary mid case US $7.8 MgCO_2_e	16,629 (55)	2,495,980	9,160,247	$71,449,923
Voluntary high case US $15 MgCO_2_e	18,999 (63)	2,887,380	10,596,685	$158,950,269
Full compliance US $30 MgCO_2_e	20,859 (69)	3,449,755	12,660,601	$379,818,026

## Discussion

### REDD+ financial viability

Floodplains topographically vary in their suitability for oil palm [23]. Understanding fine-scale landscape economics of land uses that drive deforestation, such as oil palm, is vital when assessing REDD+ financial viability in seemingly high opportunity cost landscapes such as floodplains. Our results demonstrate that in certain scenarios REDD+ could be financially competitive against oil palm development in certain scenarios. Indeed, areas with low suitability for oil palm had negative net present value (US $-109/ha), due to flood induced palm mortality [53]. In these areas, all carbon price scenarios under REDD+ had higher (positive) NPV regardless of the carbon class and the accounting stance.

In areas with medium oil palm suitability (10.96 tFFB/ha^-yr^) the lowest carbon price (US $3 MgCO_2_e) in the upfront payment accounting stance could outcompete oil palm NPV in high carbon areas only (≥350 MgC/ha). High carbon forests however, are becoming sparse in Southeast Asia due to widespread logged regimes [54]. The medium and upper voluntary market prices (US $7.8 MgCO_2_e and US $15 MgCO_2_e) were competitive in areas with ≥150 MgC/ha for the upfront payment accounting stance, although only for the US $15 MgCO_2_e for the staggered payment accounting stance. Increasingly tropical forests in Southeast Asia have lower above-ground carbon biomass due to human-practices such as selective logging [21]. For example, Malaysia’s national average carbon stock is around 164–196 MgC/ha [55]. As a result, REDD+ could be financially viable throughout Malaysia in those areas of medium oil palm suitability. Additionally, REDD+ may be a better finance alternative for smallholders who often struggle to produce good oil palm yields, especially in marginally suitable areas [8]. For example, in general Malaysia average smallholder yields are relatively high e.g. 17 tFFB/ha^-yr^ [56]; however, some studies have documented low yields e.g. 9.47 tFFB/ha^-yr^ in deep peat areas for example [57]. In Indonesia however, yield estimates for smallholders are lower averaged at 14 tFFB/ha^-yr^ [8, 58], yet variable and can be less e.g. 7.9 tFFB/ha^-yr^ [59]. Retaining natural forest can also provide a number of benefits to local people, not least in maintaining existing (traditional) livelihoods (which may provide revenue or be subsistence based), ensuring water security, as well as safeguarding forests of cultural or spiritual values, which would otherwise be lost if converted to oil palm [34]. On the more theoretical end, a carbon price of a compliance based market using US $30 MgCO_2_e, would in marginally suitable areas outcompete oil palm in regions with ≥75 MgC/ha in both accounting stances, though such a scenario is likely out of reach until international agreements are made with regards to UNFCCC REDD+.

In high oil palm suitability (full stand) areas however, REDD+ could only out-compete when: the carbon price was US $15 MgCO_2_e in areas with ≥350 MgC/ha; or, when there was a compliance based carbon price (US $30 MgCO_2_e) in areas with ≥250 MgC/ha. Such findings align with other studies that question the ability of non compliance-based markets to outcompete with high oil palm opportunity costs [14]. Malaysia and Indonesia aim to raise national oil palm yield averages to help meet national economic targets as countries strive to become high-income nations, with agricultural policies formed to assist especially smallholders [56]. Increase in palm oil efficiency, will likely elevate already high opportunity costs which may mean even further doubt as to REDD+ viability in these regions. Synergising REDD+ with other schemes such as Payments for Ecosystem Service (PES) for hydrological benefits, or biodiversity conservation through bio banking, for example [60, 61], may be a necessary way forward to compete with rising agricultural profits.

Our coarse-level analyses supported the notion that REDD+ is not competitive with oil palm within our given case study region. In this regard, it may be unlikely that REDD+ funds are targeted to regions of perceived high opportunity costs either through policy and planning level decisions within government, or through investor levels. If REDD+ funds are targeted to such areas however, landholders may refrain from entering a REDD+ contract or may contract break if they perceive to be potentially losing out on more profitable opportunities [62]. As a result, the implications of employing coarse-scale approaches for financially assessing REDD+ could be great and a more nuanced approach is needed to better understand the potential opportunities within a given landscape in order to try and reconcile biodiversity conservation and development agendas.

### Targeting REDD+ at a landscape level

Only through the fine-scale analyses was REDD+ competitive in certain regions of the floodplain. At a landscape level, REDD+ with a low carbon offset price (US $3 MgCO_2_e) in both accounting stances could outcompete oil palm across 55% (16,628 ha) of the unprotected forest; avoiding around 9 million tons of carbon dioxide emissions. These areas could be protected relatively cheaply, e.g. in the region of US $27 million for the Kinabatangan case study for 25 years. Cost-effective targeting of REDD+ projects could be considered, to enable wider conservation impacts from limited financial resources (e.g. conservation triage) [63]. In floodplain regions that experience systematic flooding events, this could provide a win-win scenario, synergising development with biodiversity conservation as floodplains generally have high levels of biodiversity [22, 64], and are large carbon sinks of above- and below-ground biomass, as well as having high carbon sequestration capacity [39, 65]. Other tropical floodplains undergoing similar patterns of forest conversion to oil palm, such as the Amazon and Congo Basins [66–67], can have extensive areas that experience inundation. For example, one wetland forest type (out of seven) in the central Amazon, was estimated to flood >200,000 km^2^ for up to 210 days per annum [68]. In the Congo Basin of the Democratic Republic of Congo, inundation and flood prone areas were estimated to cover 102,000 km^2^ [69]. These are vast areas of low suitability for oil palm, could be targeted for REDD+ using transaction funds on the existing voluntary carbon markets, providing an immediate option for governments and NGO’s.

REDD+ projects funded through the voluntary markets have been gaining momentum [48]. Although, tracking financial flows on the voluntary carbon markets is difficult, US $8 billion was noted through voluntary carbon funds and bilateral funds (e.g. the Norway-Indonesia REDD+ deal that totalled US $1 billion) [70] and an annual turnover has been estimated at US $237 million in 2011, US $216 million in 2012 possibly increasing to US $2.3 billion by 2020 [48]. If this unfolds, then these markets may provide viable opportunities for carbon-based projects across landscapes; although, the likelihood of this is under question [9]. For the other landscape level models developed in this study, between US $72 million to US $82.6 (depending on accounting stance) would be needed under the mid-range carbon offset price of US $7.8 MgCO2e, which would safeguard 55% to 63% of the unprotected forest, respectively (Table 4). Whereas for the higher voluntary price (US $15 MgCO_2_e) between US $159 million US $188 million could capture 63%, to 68% of the unprotected forest, respectively (Table 4). In the landscape however, even at the compliance price (US $30 MgCO_2_e) only 73% of the forest had higher NPV under a REDD+ mechanism than if converted to oil palm, requiring a significant amount of US $416 million. If, the proposed UNFCCC compliance-based mechanism mobilises, payments to developing countries to safeguard their forests could be in the region of US $10–60 billion per year in [71]; with more recent commitments (in Copenhagen) develop made by developed countries to provision US $100 billion a year by 2020 [72]. This study contributes much needed information to the oil palm-REDD+ debate by demonstrating that in certain contexts REDD+ can be competitive against oil palm agriculture. Nevertheless, in areas with high oil palm opportunity cost the brutal reality is that even with a high carbon price REDD+ is likely to not be financially competitive.

### Implementation issues for REDD+

Defining policies, legal frameworks and an overall governance architecture for REDD+ establishment and management is challenging, for instance, understanding roles of actors (at national, sub-national, district, and local levels, government and private), as well as pathways for payment structures for REDD+ [73–74]. This is above and beyond other technical components of REDD+ implementation e.g. monitoring, reporting and verification (MRV), social and environmental safeguard developments, and capacity building [75], needed across scales [76]. Malaysia has made headway by undertaking ‘REDD+ Readiness’ at a national level [24], and is currently implementing an EU-REDD+ project at the State and local level to facilitate a governance framework and to better understand the co-benefits of REDD+ i.e., how REDD+ can help alleviate poverty in other areas and facilitate biodiversity conservation. Understanding and assessing differing governance options for REDD+ was beyond the scope of this paper, however such a framework, especially regarding equitable benefit sharing arrangements, will likely orchestrate the incentives at which a landholder will or will not decide to participate in such a scheme [77–78]. Implementation of conservation schemes in private lands is dependent upon the landholders willingness to engage and enter such mechanisms [79]; with willingness sometimes being low, jeopardising project implementation [80]. Although REDD+ permits land titles to be retained by landholders, restrictions on land use could either be an incentive e.g. deriving income for retaining valued forest; or, disincentive if landholder wants to use the land for another function. Understanding landholder perspectives in such ways will make positive steps towards planning for effective implementation of sustainability-based incentives and will hopefully decrease incidences of contract breaking and back out of such schemes [62].

For REDD+ and other similar PES schemes, lack of land tenure can also prevent implementation [81]. There has been controversy over REDD+ especially in regards to local people [82]. REDD+ implementation however, can have a positive impact, at least at project level, on land tenure rights to prevent ‘land grabbing’ from outsiders and loss of user rights by local communities [81]. For the case study however, current land ordinance for alienated forested land (under smallholders and commercial titles) fail to provide land tenure if full cultivation is not achieved [83]. These policies restrict landholders who may wish to retain forest for financial reasons such as entering into a REDD+ scheme, or for cultural heritage, biodiversity conservation, provisioning of forest based services, for example [34]. These policies may have particularly severe implications for small scale farmers who have unsuitable agricultural land for oil palm and may face the dilemma of land seizure by government if land is not cultivated or financial loss if they invest in cultivation [23]. Moreover, these policies ensure that contributions towards national UNFCCC commitments cannot be achieved in private lands and may contentiously target REDD+ funds to production forests and protected areas rather than private land targeted for oil palm, which may not shift the *status quo* of forest conversion in private areas in Sabah.

### Methodological strengths, limitations and caveats

Models developed in this study used a conservative approach in estimating forest carbon stock and REDD+ NPV. The lack of below-ground and more comprehensive above-ground carbon stock estimates within the carbon calculations, and lack of time-averaged forest carbon storage estimates over the 25 year period, likely grossly undervalued the landscapes carbon stock [39, 84]. This maybe especially pertinent for the mangrove systems that have large underground carbon sinks [85]. Although, data could have been sourced and surrogate data used, there can be much uncertainty in below-ground carbon [40]. Moreover, this study aimed to move forward a nuanced and empirical approach to a fine scale, regional study, needed in the oil palm—REDD+ debate, as a result general data were not considered. Additionally, we acknowledge potential limitations in the use of optical data (especially NDVI) in discerning high levels of biomass [86]. However, within our CART classification we used various indices/transformations etc developed by a higher and lower resolution imagery to ensure good discrimination between the carbon classes.

In the REDD+ models, REDD+ NPV estimates were calculated using a mid-point carbon stock value whereas for the oil palm NPV, the upper range of palms per areas were used, adding in a cautionary approach to assessing REDD+ financial viability with oil palm. The REDD+ models within this study, take a conservative approach to estimating regional NPV for REDD+. If REDD+ is to be considered for the area, more comprehensive studies on above-, and below-ground carbon, and estimates for carbon sequestration over the REDD+ timeframe could see REDD+ being more competitive in the region, and elsewhere with similar contexts.

In the oil palm models, timber revenue was not incorporated into the NPV, unlike other studies [14]. This is because extensive logging in the region has depleted the lowland forest types of commercially valuable dipterocarp species [87]. In Sabah (e.g. the UNDP-GEF Project), and arguably elsewhere in Malaysia, decades of logging have rendered much of the production forests as commercially unviable with these areas now prone to conversion to oil palm, where higher opportunity costs occur [88–89]. The oil palm NPV models in this study did not include timber revenues, as these would likely be minimal. Additionally, oil palm models used sale of FFB and not CPO, as to include a context for plantations and smallholders. Models, for crude palm oil (CPO) may vary to the ones presented here, as other products such as palm kernel could likely increase profitability [51]. However, costs of production of CPO and palm kernel, at the mill level is substantial and fine scale assessments of this was beyond the scope of this study [90].

Despite the above caveats, we present a fine-scale approach to financially assessing REDD+ competitiveness with oil palm that is translatable to other regions, not least other floodplains. In applying this method elsewhere, spatial data on forest types and their suitability for oil palm e.g. degree of flooding, would be required to allow the classification of predicted suitability of areas for oil palm. Carbon stock data would be required however, if fine-scale data is unavailable, open access data such as that developed by Baccini et al. [91] could be re-classified into appropriate classes to capture variable carbon stock within the landscape. The oil palm and REDD+ economic models developed are appropriate to other regions, however parameters could and should be amended to capture a more regionalised context if that is available.

### Conclusion

Insights from this study reach well beyond its geographical remit. Southeast Asian lowland logged forests are targeted for oil palm expansion [92]. Oil palm expansion is further driving forest loss across the global tropical lowlands such as the Congo and Amazonian Basins [66–67, 93]. Indonesia alone aims to contribute a further 9 million ha of oil palm (double its current extent) [94]. These regions will include floodplains that experience similar inundation patterns and topographical constraints for oil palm as demonstrated by this case study. In these areas REDD+ may financially outcompete oil palm and provide economic rational and incentives to landholders and governments to retain these forests of high biodiversity and ecosystem services values.

## Supporting Information

S1 File REDD+ economic models. Table A REDD+ economic model for 25 MgC/ha class; Tables B REDD+ economic model for 75 MgC/ha class; Tables C REDD+ economic model for 150 MgC/ha class; Tables D REDD+ economic model for 250 MgC/ha class; Tables E REDD+ economic model for 350 MgC/ha class; Tables F REDD+ economic model for 450 MgC/ha class; and, Tables G Coarse-scale models; Tables H sensitivity analyses at 5%, 8%, 11% and 14%, for each carbon classes mid-point MgC value at a hectare level.
(XLSX)

S2 File Oil palm new planting economic models. Table A Full stand at 100% palm capacity, annually discounted at 11%; Table B Underproductive at 50% at 50% palm capacity, annually discounted at 11%; Table C Underproductive at ≤25% at 25% palm capacity, annually discounted at 11%.
(XLSX)

S3 File Carbon classification accuracy assessment and map. Table A Error matrix for the CART analysis; and, Figure A Carbon map developed from the CART classification.
(DOCX)
