# Supplementary material for: Identifying Where REDD+ Financially Out-Competes Oil Palm in Floodplain Landscapes Using a Fine-Scale Approach
Source: PLoS One. 2016 Jun 8;11(6):e0156481. doi: 10.1371/journal.pone.0156481 (PMC4898686; doi:10.1371/journal.pone.0156481)
Supplement: S3 File — Table A Error matrix for the CART analysis; and, Figure A Carbon map developed from the CART classification. (DOCX) [file pone.0156481.s003.docx]

**Supporting Information**

**File S3: Carbon classification accuracy assessment and map**

**REDD+ financially out-competes oil palm cultivation in floodplain forest systems in Borneo**

Nicola K. Abram*^;^ Douglas C. MacMillan; Panteleimon Xofis; Marc Ancrenaz; Joseph Tzanopoulos; Robert Ong; Lian Pin Koh; Benoit Goossens; Christian Del Valle; Lucy Peter; Alexandra C Morel; Isabelle Lackman; Robin Chung; Harjinder Kler^;^ Laurentius Ambu; William Baya; Andrew T. Knight.

*Corresponding author ([nicola_abram@hotmail.com](mailto:nicola_abram@hotmail.com))

**Table S3A** **Error matrix for the CART analysis**

| Carbon classes (MgC) | <50 | 50-100 | 100-200 | 200-300 | 300-400 | >400 | sum |
| --- | --- | --- | --- | --- | --- | --- | --- |
| Class 1: <50 | 12 | 0 | 0 | 0 | 0 | 0 | 12 |
| Class 2: 50-100 | 0 | 10 | 1 | 1 | 1 | 0 | 12 |
| Class 3: 100-200 | 0 | 0 | 40 | 5 | 0 | 0 | 45 |
| Class 4: 200-300 | 0 | 0 | 4 | 23 | 1 | 0 | 28 |
| Class 5: 300-400 | 0 | 0 | 2 | 0 | 12 | 0 | 14 |
| Class 6: >400 | 0 | 0 | 0 | 0 | 0 | 3 | 3 |
| Unclassified | 0 | 0 | 0 | 1 | 0 | 0 | 1 |
| sum | 12 | 10 | 47 | 29 | 14 | 3 | 115 |
| Correctly classified | 100.0 | 100.0 | 85.1 | 79.3 | 88.9 | 100.0 |  |

The error matrix for the Kinabatangan classification was based on the 115 testing objects. The error confusion matrix shows Class 1 (<50 MgC ha); Class 2 (50-100 MgC ha) and Class 6 (>400 MgC ha) were classified perfectly i.e. classes achieved 100% agreement with 12, 10 and 3 training objects respectfully. Whilst the remaining classes achieved excellent accuracy of 88.9% for Class 5 (300-400 MgC ha); 85.1% for Class 3 (100-200 MgC ha); and 79.3% for Class 4 (200-300 MgC ha) with 14, 47 and 29 training objects respectfully.

Inevitably inherent sources of error are likely to have occurred and although impossible to eradicate should be acknowledged. Sources of error include: (1) biases in plot data sampling towards certain habitats; three habitats had one hectare only and two habitats (Nipah palm and Swamp) had no data; (2) allometric calculations used to convert botanical data into carbon estimates, were restricted to equations that use diameter at breast height (DBH) and not height, and only trees with ≥10 DBH; (3) Spatial representation of plots in the GIS was difficult for line transects (of 20 m width); and (4) Spatial extrapolation (up-scaling) from 0.5 ha plot data (smaller) to overlapping ‘sample objects’ (larger) that assumed object was homogenous; and spatial extrapolation.

**
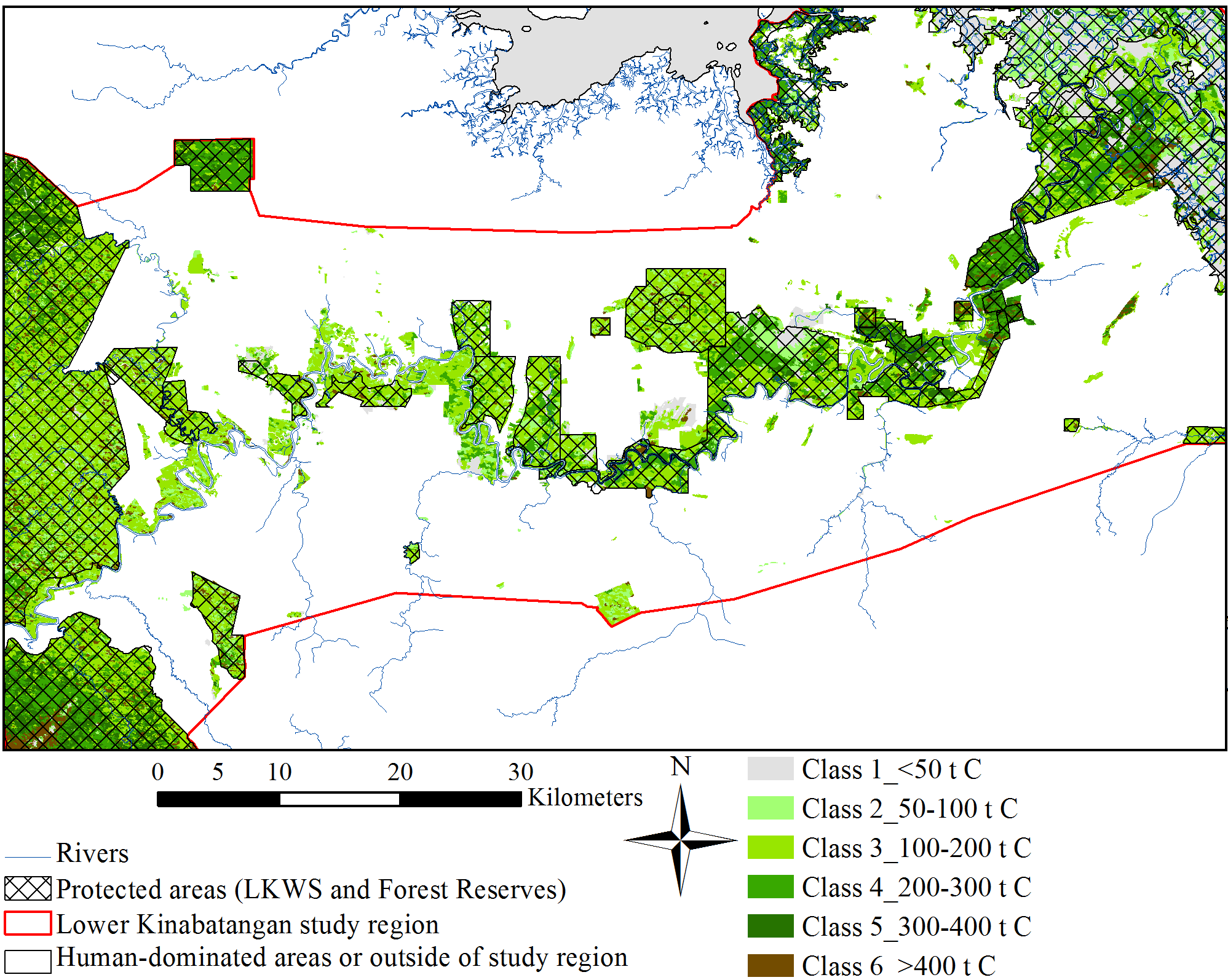
**

**Figure S3A:** **Carbon map developed from the CART classification.** Spatial distribution of carbon classes in all forested regions of the Lower Kinabatangan study area predicted from the Classification and Regression Tree (CART) analysis (in eCognition); along with Protected Area network (black outline); Lower Kinabatangan study region (red outline) and Sabah outline (grey).
